# Complete genome sequence of *Sporosarcina* sp. GTC18466 isolated from a snake in Japan

**DOI:** 10.1128/mra.01172-25

**Published:** 2026-01-20

**Authors:** Masahiro Hayashi, Jun Yonetamari, Yoshinori Muto, Kaori Tanaka

**Affiliations:** 1 Institute for Glyco-core Research iGCORE, Gifu University, Gifu, Japan; 2 Division of Anaerobe Research, Life Science Research Center, Gifu University 201111, Gifu, Japan; 3 Gifu University Center for Conservation of Microbial Genetic Resource, Gifu, Japan; 4 Division of Clinical Laboratory, Gifu University Hospital 476117, Gifu, Japan; 5 United Graduate School of Drug Discovery and Medical Information Sciences, Gifu University 520344 , Gifu, Japan; University of Wisconsin-Madison, Madison, Wisconsin, USA

**Keywords:** *Sporosarcina*, genomes

## Abstract

The genus *Sporosarcina* belongs to the family Caryophanaceae within the class Bacilli. Here, we report the complete genome sequence of a *Sporosarcina* species obtained from a snake skin in Japan. The genome comprised a circular chromosome measuring 3,223,418 bp in length.

## ANNOUNCEMENT

Identifying pet animal pathogens through genomic analysis is important for understanding pathogenic mechanisms and zoonotic infection risks (1, 2). *Sporosarcina* species have frequently been isolated from animals and humans (3, 4). We analyzed strain GTC18466, isolated from the surface swab of the skin of a healthy *Python regius* that was kept at a pet shop in Seki City, Gifu, Japan, in 2024. The strain was cultured on tryptic soy agar with sheep blood at 37°C for 48 h under aerobic conditions and was submitted to our laboratory as an unknown strain for further identification. The isolate was cultured and purified twice under the conditions described above. The strain was stored at –80°C after four passages during identification.

The strain was cultured on tryptic soy agar under the same conditions. Colonies were scraped, suspended in TE buffer, and pelleted by centrifugation. Genomic DNA was extracted using the NucleoBond HMW DNA kit (MACHEREY-NAGEL, Japan) according to the manufacturer’s instructions.

For identification, the 16S rRNA gene was sequenced after PCR amplification using primers 16S-8F (AGAGTTTGATCMTGGCTCAG) and 16S-1492R (GGYTACCTTGTTACGACTT). BLASTN analysis (5) identified the strain as *Sporosarcina* sp., most closely related to *Sporosarcina koreensis* F73 (Type strain accession no. DQ073393), with 98.5% similarity using phylogenetic analysis.

The genome was sequenced using PromethION 2i (Oxford Nanopore Technologies [ONT], Oxford, UK) for long-read sequencing and DNBSEQ (MGI Tech Co., Ltd., Shenzhen, China) for short-read sequencing (6–8). The same DNA extract was used for both sequencing processes. A library was constructed with a Native Barcoding Kit (SQK-NBD114-24; ONT) without shearing for long-read sequencing. Small DNA fragments were removed using the Short Read Eliminator XS kit (Pacific Biosciences of California, Inc., USA). Sequencing was performed using a PromethION 2i (ONT) with a FLO-PRO114M flow cell. Base calling was performed with Dorado v.7.2.14 (ultra-high accuracy mode), and raw reads were trimmed and quality-filtered using NanoFilt v.2.7.1 (9) with parameters “-l 1,000 -q 10 –headcrop 50.” For short reads, library construction was done with the MGIEasy FS DNA Library Prep Set (MGI Tech), and 2 × 150 bp paired-end sequencing was performed on the DNBSEQ-G400 platform (MGI Tech). Reads were processed with fastp v0.20.1 (10) using “-q 30 -n 20 -t 1 -T 1.” Short-read quality was evaluated using fastp (10), and long-read quality was scored using NanoPlot v1.32.1 (0). Short reads (with over 96.8% of bases >Q30) and long reads (mean read quality of 16.0) were assembled using Unicycler v.0.4.8 (11) with default settings. Assembly circularization and rotation were performed automatically in Unicycler, and the assembly was rotated to start with *dnaA* on the forward strand. The contig graph was visualized with Bandage v0.8.1 (12), and the taxonomic integrity was confirmed using Blobtools v1.0 with short reads (13).

Table 1 summarizes the genomic information. Unicycler and Bandage indicated a complete circular genome. Quality and genome statistics were computed using CheckM (v1.2.2) (14) and seqkit (v2.9.0) (15). CheckM confirmed that the genome was 100% complete, with 0% contamination. The DDBJ Fast Annotation and Submission Tool v.1.2.0 (16) predicted 3,182 coding sequences, 27 rRNAs, 70 tRNAs, and 1 CRISPR sequence. Species identification using an average nucleotide identity value of the genome sequence with GTDB-tK (v.2.3.2) was unsuccessful (17). Furthermore, the taxonomic identification of the strain GTC18466 was performed using the Type Strain Genome Server (18). Genome- and rRNA-based comparisons identified *Sporosarcina* as the most closely related genus; however, no species exceeded the species delineation threshold in the digital DNA–DNA hybridization value.

**TABLE 1:** Information regarding the complete genome sequence of a *Sporosarcina* sp. strain isolated from a snake in Japan

	Strain name
Parameter	*Sporosarcina* sp. GTC18466
DNBSEQ sequencing^*a*^	
No. of reads	4,751,686
Size (kb)	693,264
Avg coverage (×)	227
DRA accession no.	DRR726413
ONT sequencing^*a*^	
No. of reads	128,640
Size (kb)	1,378,822
Avg read length (bp)	10,719
Avg coverage (×)	428
N_50_	16,091
DRA accession no.	DRR726414
Assembly	
Assembly N50 (bp)^*c*^	3,223,418
Estimated genome completeness (%)^*c*^	100.0
Estimated genome contamination (%)^*c*^	0.0
Genome structure	One chromosome
DDBJ/GenBank accession no.	AP044037
Genome size (bp)	3,223,418
GC content (%)	
(chromosome/plasmid name)	50.5
Average read depth	
	226.2
No. of coding sequences^*b*^	3,182
Number of rRNAs^*b*^	27
Number of tRNAs^*b*^	70
Number of CRISPRs^*b*^	1

^*a*^ DRA, DDBJ Sequence Read Archive.

^*b*^ DFAST, DDBJ Fast Annotation and Submission Tool.

^*c*^ Determined with seqkit v2.9.0.

## Data Availability

The genome sequences of the *Sporosarcina sp*. GTC18466 strain from the snake have been deposited in DDBJ (https://www.ddbj.nig.ac.jp/index.html) under the accession number AP044037. Raw sequence data for GTC18466 have been deposited in the DDBJ/Sequence Read Archive under accession numbers DRR726413 and DRR726414.
